# Survival and predictors of mortality among HIV-positive children on antiretroviral therapy in public hospitals

**DOI:** 10.1186/s40545-022-00448-6

**Published:** 2022-08-17

**Authors:** Yalemgeta Biyazin, Kalkidan Wondwossen, Azene Bantie Wubie, Melese Getachew, Bereket Gebremichael

**Affiliations:** 1 School of Nursing and Midwifery, College of Health Sciences, Addis Ababa University, Addis Ababa, Ethiopia; 2 Department of Pediatrics and Child Health Nursing, College of Health Sciences, Debre Markos University, Debre Markos, Ethiopia; 3 Department of Pharmacy, College of Health Sciences, Debre Markos University, Debre Markos, Ethiopia

**Keywords:** Children, Antiretroviral therapy, HIV, Survival, Ethiopia

## Abstract

**Background:**

Human immunodeficiency virus and acquired immunodeficiency syndrome had created enormous challenges worldwide, and continues to be the world’s serious health and development challenges. Globally, at the end 2017, there were 1.8 million children (< 15 years) living with HIV. The survival of HIV-positive children treated with ART depends on a variety of factors, which might vary greatly with economic, socio-demographic, behavioral risk, and health factors. This study aimed to assess survival status and predictors of mortality among HIV-positive children on antiretroviral therapy at East Gojjam Zone Public hospitals, Northwest Ethiopia.

**Methods:**

An institution-based retrospective cohort study was conducted in selected hospitals of the East Gojjam zone, Northwest Ethiopia, among < 15-year-old children who were newly enrolled in HIV care clinic from January 1st, 2014 to December 31, 2018. Data were collected from patient charts from March 1 to 22, 2019 using a standardized checklist. Data were analyzed by SPSS version 24. A Kaplan–Meier curve and log-rank test were used to estimate the survival time and compare survival curves between variables. Multivariable Cox proportional-hazards model was fitted to identify predictors of survival status taking p-value < 0.05 as statistically significant.

**Result:**

In this study, a total of 251 HIV-positive children on ART were followed up for a total of 60 months, with a mean survival time of 55.54 (± 0.83) (95% CI: 53.90–57.17) months. The overall mortality incidence rate in the cohort during the 626 Child-Year-Observation (CYO) was 2.56/100 CYO. The overall estimated survival probability after starting ART was 0.90 at 60 months of follow-up. In this study age < 5 years, Hgb < 10 gm/dl, CD4 count below threshold, cotrimoxazole preventive therapy, and subnormal weight for height were statistically significant predictors of survival status (*P* < 0.05).

**Conclusion and recommendation:**

Age, hemoglobin level, CD4 count, weight for height, and not taking cotrimoxazole preventive therapy were independent predictors of mortality. Therefore, concerned stakeholders should focus on the above-mentioned predictors of mortality and nutritional interventions to enhance the survival of HIV-infected children on antiretroviral therapy.

## Background

Human immunodeficiency virus (HIV) and acquired immunodeficiency syndrome (AIDS) have created enormous challenges worldwide. HIV has become one of the world’s most serious health and development challenges since the first cases were reported in 1981. According to UNAIDS (2022), 84.2 million people have become infected with HIV and 40.2 million have been died since the start of the epidemic. In 2021 there were 38.4 million people living with HIV/AIDS out of which children (< 15 years old) comprise 1.7 million cases [1, 2]. In Ethiopia, around 610,000 people were living with HIV of which 42,000 were children (0–14 ages) in 2021 [11–18 UNAIDS].

More than 90% of pediatrics HIV infection occurs through mother-to-child transmission which implies that global distribution of pediatrics HIV infection is similar with the distribution of HIV in women [4]. Infants and young children infected with HIV have exceptionally higher morbidity & mortality. Up to 52% and 75% of children die before the age of two and five years, respectively, in the absence of any intervention [6]. ART should be initiated for all children living with HIV regardless of WHO clinical stage or at any CD4 cell count [3]. However, only 52% of all children aged 0–14 years living with HIV had access to treatment globally in 2021 [1].

The vast majority of people living with HIV are located in the low- and middle-income countries. The first evidence of HIV infection in Ethiopia was detected in 1984. Since then, AIDS has claimed the lives of millions and has left behind hundreds of thousands of orphans. HIV/AIDS epidemic has remained one of the important public health challenges [4]. Ethiopia has one of the largest populations of HIV-infected people in the world [5]. Over the last several years, there has been a dramatic decline in new pediatric infections, but children born infected with HIV are in critical need of lifesaving HIV treatment.

Predictors of mortality among children on HAART in the country were inconsistently reported by fragmented studies conducted in different study areas [6–13]. The survival of HIV-positive children treated with ART depends on a variety of factors, which might vary greatly with economic, socio-demographic, behavioral risk and health factors. Even though ART has shown significant clinical importance by meeting the goal of therapy, still children were facing a number of deaths that could be avoided by appropriate interventions on certain factors such as socio-economic, demographic, treatment-related and health factors which include the child’s age, CD4 count/CD4% at ART initiation, WHO stage, hemoglobin level, ART adherence [14–20].

No study was conducted about the survival and predictors of mortality among HIV-infected children on ART in East Gojjam zone. The aim of this study was; therefore, to assess survival status and predictors of mortality among HIV-positive children on antiretroviral therapy in public hospitals of East Gojjam zone.

## Methods

### Study setting, design, and period

An institutional based retrospective cohort study was conducted at Debre Markos Referral Hospital (DMRH) and Shegaw Mota District Hospital among children less than 15 years who were living with HIV and initiated for ART from January 1st 2014 to December 31st, 2018. According to 2007 Census, East Gojjam zone has a total population of 2,153,937, of whom 1,066,716 are men, with an area of 14,004.47 square kilometers. It has a population density of 153.80, while 213,568 or 9.92% are urban inhabitants [21].

These two hospitals were purposively selected because they were the only hospitals in the study area which provide ART services at starting time of study period. Debre Markos Referral Hospital is the only referral hospital in East Gojjam zone which is found in Debre Markos town. Shegaw Mota District Hospital (SMDH) is one of primary hospitals in east Gojjam zone which is found in Mota town. According to information obtained from administrative offices of these hospitals, DMRH and SMDH serve more than 3.5 million and 1.5 million populations in their catchment area, respectively [21–23].

### Study population

All medical records of HIV-positive children who attended the ART unit of DMRH and SMDH from January 1st, 2014–December 31st, 2018 were considered for inclusion. All HIV-positive children less than 15 years old and who were newly enrolled on ART were included; whereas, children whose medical charts were not found during data collection and/or children’s chart having incomplete data on major variables of the study were excluded.

### Sample size and sampling procedure

The sample size was determined using double population proportion formula by considering the covariates. Three variables (INH prophylaxis, baseline CD4 count, and hemoglobin level) that were reported as predictors of mortality among children on ART based on study done in Arba Minich, Ethiopia [8] were used to calculate sample size. When calculated with Epi info version 7 statistical package, baseline CD4 count below the threshold (P_1 =_ 20.99 and P_2 =_ 11.25) gave the maximum sample size (486). Percentage in exposed (*P*_1_ = 20.99), percentage in non-exposed (*P*_2_ = 11.25), a confidence level of 95% (Zα/2 = 1.96), and one-to-one exposed to non-exposed ratio (r) were considered. Charts of children enrolled on ART during the study period were recruited by census as their number was small number of than that obtained from the calculated sample (486). Finally, the selected medical charts were reviewed from March 1 to 22, 2019.

### Study variables

Survival status was dependent variable of this study. The different independent variables used were socio-demographic factors (age at initiation of ART, sex, residency, primary caregiver, and caregiver religion), clinical and laboratory factors (baseline WHO stage, tuberculosis (TB) positive at baseline, CD4 count/percent at baseline, past opportunistic illness, and hemoglobin level), treatment-related factors (early/delayed initiation, baseline ART regimen, OI prophylaxis, ART adherence, and drug side effects), and nutritional factors (underweight, stunting, and wasting).

### Data collection

A data abstraction format was developed in English language from the standardized ART entry and follow-up form that is currently used by the ART clinics of the study hospitals. It contains three parts; socio-demographic information and nutritional information, clinical and laboratory information, and treatment-related information. The starting point for retrospective follow-up was the time from initiation of ART, and the end point was date of death, date of lost to follow-up, date of transfer out or date of last contact until January 31^th^, 2018. Before collecting the data, the records were reviewed (both baseline and follow-up records). Death was confirmed by death certificate complemented by registration and were identified by their medical record number. The most recent laboratory test results before starting ART were used as a baseline value. If there is no pre-treatment laboratory test, results obtained within one month of ART initiation were used as a baseline. In cases where there were two results obtained within a month, the mean value was used.

### Data quality control

To ensure quality of the data, pretest was conducted with 5% of the sample population in Finote Selam Hospital found in west Gojam Zone, Amhara regional state. Four nurses, who have been working on ART unit, participated in data collection. One supervisor in each hospital was assigned to closely supervise the entire data collection process. Training on record review was given to data collectors and supervisors for one day before actual data collection task. All completed data collection forms were examined for completeness and consistency during data management, storage, cleaning and analysis. Consistency was examined through random selection of cards by the principal investigator and cross-checked for their similarity. The overall data collection process was under control of the principal investigator. The data were entered and cleaned by principal investigator before analysis.

### Data analysis

Data were coded and then entered to EPI-data 3.5.3 and transferred to SPSS v 24 statistical software for analysis. Data exploration was undertaken to see if there are odd codes or items that were not logical and then subsequent corrections were made. The actuarial life table was used to estimate probabilities of survival after ART initiation at different time intervals. Kaplan–Meier survival curve together with log-rank test was used to check the presence of difference in survival among categories of covariates and log-rank test was used to compare survival curves. Cox regression was carried out to find predictors of survival status. Patient’s cohort characteristics were described in terms of central tendency and dispersion value for continuous data, and frequency distribution for categorical data. Finally, the outcome of each subject was dichotomized into censored or death. Bivariate Cox regression was first fitted and those independent variables which became significant on the bivariate regression having *p*-value ≤ 0.25 level of significance were included in the multivariable analysis. Cox proportional-hazard regression was fitted at 5% level of significance to determine the net effect of each explanatory variable on time to death after ART initiation (hazard ratio with its 95% confidence interval and p-values was used to measure strength of association and identify statistically significant result). *P*-value < 0.05 was considered as statistically significant association. Finally, the results of the study were presented with text, graph and table.

### Operational definitions

#### Censored

Those HIV-positive children who did not develop the outcome of interest (death) until the end of follow-up period, those lost to follow-up or transferred out to a different care unit during the study.

#### Children

A group of individuals aged less than 15 years.

#### Duration on ART

Is defined as the time between the start date of ART and the date of last contact with the health facility.

#### Event

The occurrence of death from initiation ART to the end of the study.

Medication adherence: was classified as Good (95% or ≤ 2 missed drug doses of 30 doses or < 3 missed drug doses of 60 doses); Fair (drug adherence of 85–94% or 3–5 missed drug doses of 30 doses or 4–9 missed drug doses of 60 doses); and Poor (drug adherence of < 85% or ≥ 6 doses of missed ART drug doses of 30 doses or > 9 doses missed ART drug doses of 60 doses) [24].

#### Moderate stunting

Children having height/age Z-score < −  2 SD [25, 26].

#### Moderate underweight

Children having weight/age Z-score <  −  2 SD [25, 26].

#### Moderate wasting

Children having weight/height Z-score <  −  2 SD [25, 26].

#### Severe stunting

Children having height/age Z-score <  −  3 SD [25, 26].

#### Severe underweight

Children having weight/age Z-score <  −  3 SD [25, 26].

#### Severe wasting

Children having weight/height Z-score <  −  3 SD [25, 26].

#### Survival status

In this research, survival status was defined as the outcome of patients was sourced from patient clinical data files and was dichotomized into censored or death.

### Ethical considerations

Ethical clearance letter was obtained from Institutional Review Board of School of Nursing and Midwifery, College of Health science, Addis Ababa University. Permission was obtained from Debre Markos referral hospital and Shegaw Mota district hospital. All collected data were coded to keep the confidentiality. Names and unique numbers of patients were not included in the data collection format, and the data were not disclosed to any person other than the investigators.

## Results

### Socio-demographic characteristics

In this study, a total of 251 HIV-positive children on ART were followed up for a total of 60 months (January 1st, 2014–December 31st, 2018). Among HIV-positive children (age 0–14 years), who were initiated for ART from January 1, 2014 to December 31, 2018, 264 records were reviewed. Of these, 251 (95.4%) of records were used in the final analysis, while the remaining 12 (4.6%) records were not included in the final analysis due to missing data from the files. About half (51.8%) of the study participants were males and more than half (57%) of them were from urban areas. The mean and median (SD) age of the children at ART initiation was 89.13 and 96 (± 47.5) months, respectively. The majority of study participants (82.1%) were living with their parents and nearly two-thirds (64.5%) of their caregivers were married. The majority of study participants (78.1%) were orthodox Christian religion followers (Table 1).

**Table 1:** Socio-demographic characteristics of children on ART at East Gojjam Zone public hospitals, Amhara regional state, Northwest Ethiopia, 2019

Variable	Outcome of the child		
	Death *n*(%)	Censored *n*(%)	Total *n*(%)
Age (months)			
< 12	4 (25)	15 (6.4)	19 (7.6)
12–59	6 (37.5)	47 (20.0)	53 (21.1)
60–179	6 (37.5)	173 (73.6)	179 (71.3)
Sex			
Male	6 (37.5)	124 (52.8)	130 (51.8)
Female	10 (62.5)	111 (47.2)	121 (48.2)
Residence			
Urban	11 (68.8)	132 (56.2)	143 (57.0)
Rural	5 (31.3)	103 (43.8)	108 (43.0)
Primary caregiver			
Parents	13 (81.3)	193 (82.1)	206 (82.1)
Relatives	2 (12.5)	33 (14.0)	35 (13.9)
Guardian and orphan	1 (6.3)	8 (3.4)	9 (3.6)
Religion of caregiver			
Orthodox	13 (81.5)	183 (77.9)	196 (78.1)
Muslim	2 (12.5)	42 (17.9)	44 (17.5)
Catholic	1 (6.3)	3 (1.3)	4 (1.6)
Protestant	–	7 (3.0)	7 (2.8)
Current status of parents			
Both alive	10 (62.5)	180 (76.6)	190 (75.7)
Mother alive, father died	3(18.8)	31 (13.2)	34 (13.5)
Father alive, mother died	1 (6.3)	13 (5.5)	14 (5.6)
Both died	2 (12.5)	11 (4.7)	13 (5.2)
Marital status of caregiver			
Single	1 (6.7)	21 (9.2)	22 (9.0)
Married	8 (53.3)	154 (67.2)	162 (66.4)
Divorced	5 (33.3)	31 (13.5)	36 (14.8)
Widowed	1 (6.3)	16 (7.0)	17 (7.0)
Separated	–	7 (3.1)	7 (2.9)

### Clinical, laboratory and ART information

Of the total 251 cohorts, 155 (66.80%) children started ART being in mild WHO clinical disease stage of HIV (I or II). After ART initiation, 75 (29.9%) of the children had opportunistic infection. About three-fourths (74.1%) of under-5 years children had appropriate developmental status at ART initiation while 67% of children aged 5–14 year perform their daily activity. Nearly three-fourths (73.6%) of the children had CD4 count or percent above the threshold for severe immunodeficiency. Similarly, three-fourths 72.9% of the children have been taking cotrimoxazole preventive therapy. Fifty-one (20.1%) of children were anemic at ART initiation. Majority of children 217 (86.5%) had good ART adherence (Table 2).

**Table 2:** Baseline clinical, laboratory and ART information of children on ART at East Gojjam zone public hospitals, Amhara regional state, Northwest Ethiopia, 2019

Variable	Outcome of the child		
	Death *n*(%)	Censored *n*(%)	Total *n*(%)
Baseline WHO stage			
Stage I	3 (18.8)	84 (35.7)	87 (34.7)
Stage II	3 (18.8)	65 (27.7)	68 (27.1)
Stage III	5 (31.3)	61 (26.0)	66 (26.3)
Stage IV	5 (31.1)	25 (10.6)	30 (12.0)
CD4 count at baseline			
Below threshold	12 (75)	174 (77.0)	178 (73.6)
Above threshold	4 (25)	40 (17.2)	51 (20.5)
Hemoglobin level			
< 10 mg/dl	11 (68.8)	40 (17.2)	51 (20.5)
≥ 10 mg/dl	5 (31.2)	193 (82.8)	198 (79.5)
Functional status for age ≥ 5 years			
Working/functional	2 (28.57)	130 (68.4)	132 (67.0)
Ambulatory	1 (14.29)	53 (27.9)	54 (27.4)
Bedridden	4 (57.14)	7 (3.7)	11 (5.6)
Developmental status at baseline for age < 5 years			
Appropriate	6 (66.67)	34 (75.6)	40 (74.1)
Delayed	2 (22.22)	7(15.6)	9 (16.7)
Regressed	1 (11.11)	4(8.9)	5 (9.3)
Cotrimoxazole prophylaxis			
Yes	5 (31.25)	178 (75.7)	183 (72.9)
No	11 (68.75)	57 (24.3)	68 (27.1)
Opportunistic infections			
Yes	10 (62.5)	65 (27.7)	75 (29.9)
No	6 (37.5)	170 (72.3)	176 (70.1)
ART adherence			
Good	8 (50)	209 (88.9)	217 (86.5)
Fair	2 (12.5)	17 (7.2)	19 (7.6)
Poor	6 (37.5)	9 (3.8)	15 (6.00)
Drug side effect			
Yes	4 (25)	57(24.3)	61 (24.3)
No	12 (75)	178(75.7)	190 (75.7)
The regimen changed			
Yes	5 (31.25)	10 (4.3)	15 (6.0)
No	11 (68.75)	225 (95.7)	236 (94.0)
Weight for height			
Normal	6 (37.5)	178 (75.7)	184 (73.3)
Moderate wasting	4 (25.0)	52 (22.1)	56 (22.3)
Severe wasting	6 (37.5)	5 (2.1)	11 (4.4)
Height for age			
Normal	6 (37.5)	171 (72.8)	177 (70.5)
Moderate stunting	6 (37.5)	56 (23.8)	62 (24.7)
Severe stunting	4 (25.0)	8 (3.4)	12 (4.8)
Height for age			
Normal	6 (37.5)	165 (70.2)	171 (68.1)
Moderate underweight	6 (37.5)	59 (25.1)	65 (25.9)
Severe underweight	4 (25.0)	11 (4.7)	15 (6.0)

**Fig. 1:**
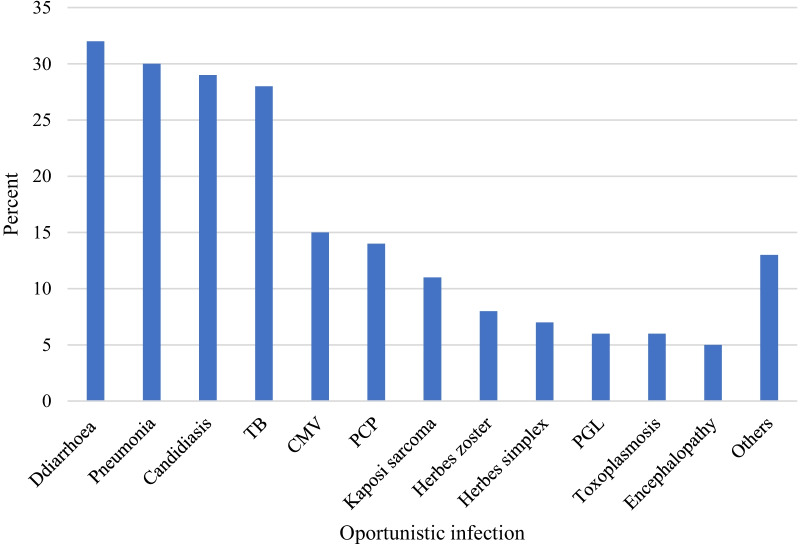
Baseline opportunistic infection of HIV-positive children on ART in East Gojjam zone public hospitals Amhara regional state, Northwest Ethiopia, 2019

Regarding baseline opportunistic infection, diarrhea was the most prevalent infection encountered by 32 (12.5%) participants. Pneumonia (11.55%) and candidiasis (11.95%) were the second and third most common opportunistic infections, respectively (Fig. 1).

**Fig. 2:**
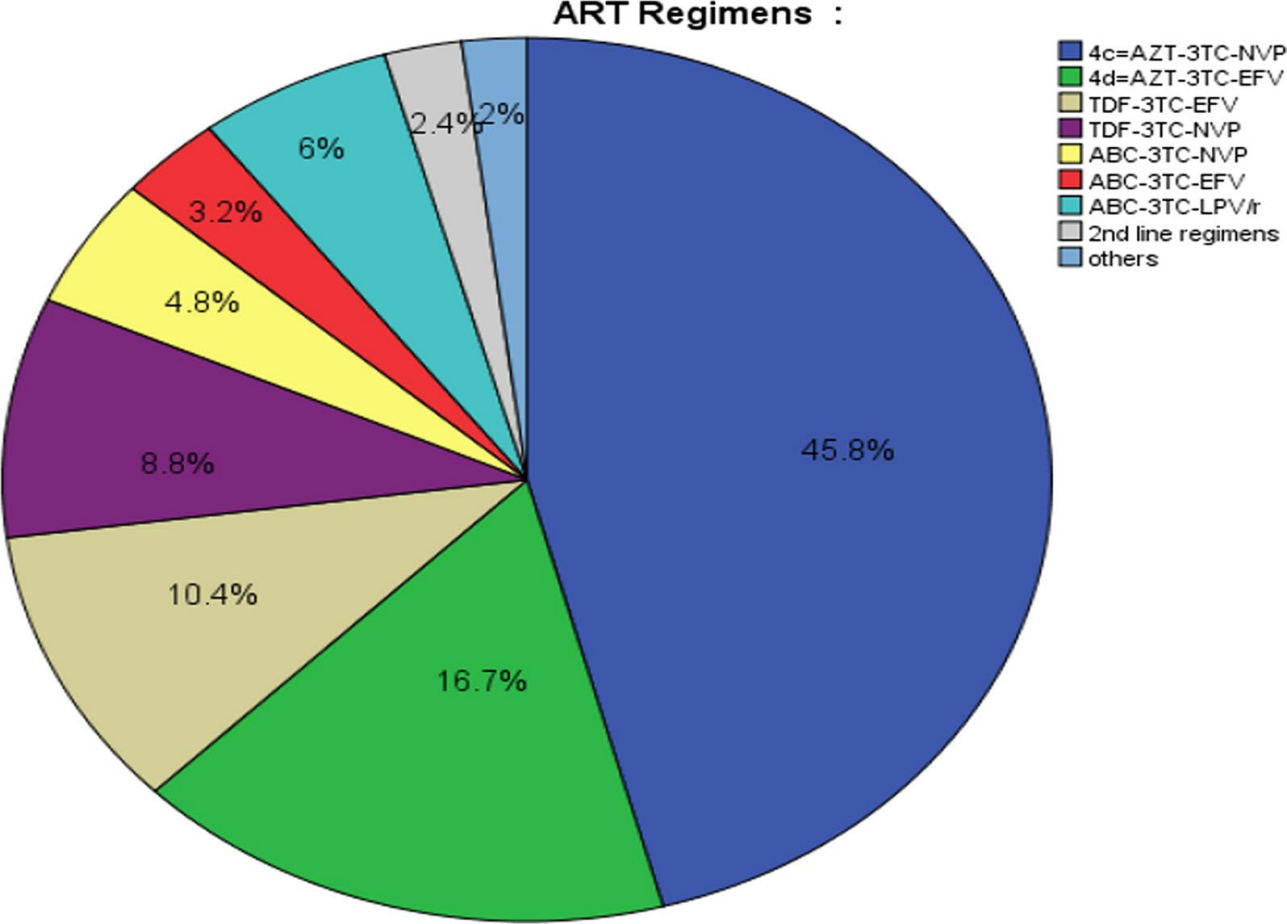
Baseline ART regimen given for HIV-positive children on ART at East Gojjam zone public hospitals Amhara regional state, Northwest Ethiopia, 2019

Regarding ART regimen, 115(45.8%) of children have taken a drug of AZT-3TC-NVP followed by 42(16.7%) of children have taken a drug of AZT-3TC-EFV (Fig. 2). In this study, the nutritional status of the study participant showed that, 67(26.3%) were wasted, 72(29.5%) were stunted and 80 (31.9%) were underweight (Table 2).

### Survival status of children on ART

**Fig. 3:**
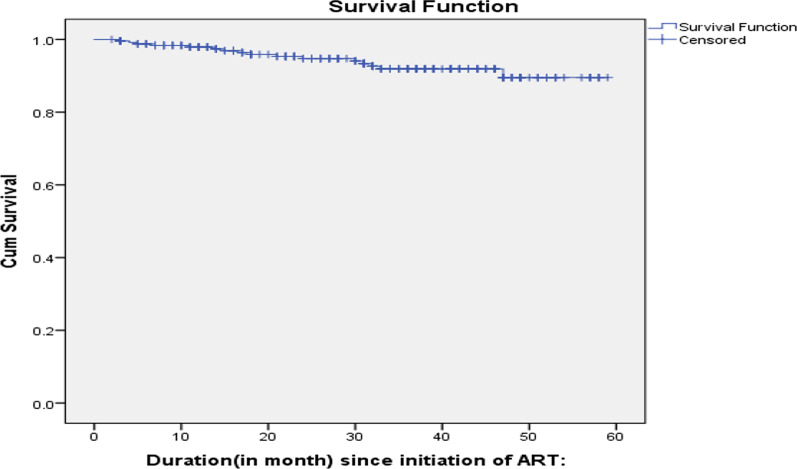
The overall Kaplan–Meier survival curve with 95% confidence intervals of children on ART at East Gojjam zone public hospitals Amhara regional state, Northern Ethiopia, 2019

In this study, a total of 251 HIV-positive children on ART were followed up for a total of 60 months, with a mean (SD) survival time of 55.54 (± 0.83) months (95% CI:53.90–57.17). The overall mortality incidence rate in the cohort during the 626 child-year-observation (CYO) was 2.56/100 CYO. Kaplan–Meier estimation of survival showed that overall estimated survival after starting ART was 90% at 60 months of follow-up. The estimated cumulative survival was 0.98, 0.96, 0.92, and 0.90 at 12, 24, 36, and 60 months, respectively. Sixteen (6.37%) patients died in the study period, but 235 (93.62%) were censored till the end of the study. Among these, 199 (79.3%) were alive, 16 (6.37%) were lost to follow-up, and 20 (8%) were transferred to other health facilities. On the Kaplan–Meier survival curve for time to death of the child on ART, the probability of survival decreases as the follow-up time increases. This study showed that the highest rate of mortality was encountered between 30 and 36 months after initiation of ART (Fig. 3).

### Survival time of children on ART

In this retrospective cohort study, the mean survival time for the younger children had a shorter survival time than those of older children. The mean (SD) survival time for children aged less than one year were 39.12 (± 4.25) months with 95% CI:30.80–47.45, and the mean (SD) survival time for 5–14 years age group was 57.27 (± 4.69) with 95% CI:55.92–58.63 months. This difference was statistically significant with *p*-value < 0.001.

The mean (SD) survival time for those who have been taking cotrimoxazole preventive therapy (CPT) was found to be 57.47(± 0.68) months as compared to those who were not taking cotrimoxazole preventive therapy with a mean (SD) survival time of 51.06 (± 1.93) months. This difference is statistically significant with *p*-value = 0.003.

Study participants who had a low hemoglobin level (< 10 gm/dl) have lower survival time as compared to those who had a high hemoglobin level (≥ 10 gm/dl). The mean (SD) survival time for those having a low hemoglobin level was 47.20 (± 2.84) months and the mean (SD) survival time for those having hemoglobin level ≥ 10 gm/dl was 57.56 (± 0.64) months. The survival time difference between the groups was found statically significant with P-value < 0.0001.

The mean (SD) survival time for those who had CD4 count below the threshold at baseline was 48.15 (± 2.47) months and 95% C/I 43.32–53.00, but it was 57.79 (± 0.60) months and 56.61–58.96 for those who had CD4 count above the threshold at baseline. This difference was statistically significant with p-value < 0.0001 (Table 3).

**Table 3:** Survival time, significance and log-rank test for the study population for different characteristics of children during 5 year of follow-up (Kaplan–Meier method) of children on ART at East Gojjam zone public hospitals, Northwest Ethiopia, 2019

Covariates	Survival time per month, (95% CI)		Log-rank test (*p*–value)
	Mean ± SE	95% (CI)	
Age in month			
< 12	39.12 ± 4.25	30.80–47.45	19.54 (0.000)
12–59	50.31 ± 2.52	45.38–55.25	
≥ 60	57.27 ± 4.69	55.92–58.63	
Sex			
Male	55.64 + 0.94	53.80–57.48	2.08 (0.149)
Female	54.38 + 1.38	51.67–57.09	
Residence			
Urban	54.98 ± 1.16	52.70–57.25	0.87 (0.351)
Rural	55.48 ± 1.09	53.33–57.62	
CD4 count			
Below threshold	48.15 ± 2.47	43.32–52.20	21.90(< 0.001)
Above threshold	57.79 ± 0.60	56.61–58.96	
Hemoglobin level			
< 10 mg/dl	47.20 ± 2.84	41.63–52.76	25.23 (< 0.001)
≥ 10 mg/dl	57.56 ± 0.64	56.31–58.81	
WHO HIV stage			
Stage I	57.04 ± 1.11	54.87–59.21	10.23 (0.017)
Stage II	55.85 ± 1.22	53.47–58.23	
Stage III	54.31 ± 1.57	51.23–57.40	
Stage IV	42.97 ± 3.18	36.73–49.21	
Functional status ≥ 5 years			
Working/functional	58.20 ± 0.56	57.11–59.30	46.96 (< 0.001)
Bedridden	37.50 ± 5.14	27.42–47.58	
Ambulatory	57.12 ± 0.87	55.41–58.83	
Developmental status (< 5yrs)			
Appropriate	44.49 ± 2.80	39.00–49.98	603 (0.819)
Delayed	36.25 ± 3.28	29.83–42.67	
Regressed	24.50 ± 4.60	15.49–33.51	
Weight for height			
Normal	57.22 ± 0.71	55.82–58.62	17.02 (0.000)
Moderate wasting	52.57 ± 2.08	48.49 -56.64	
Severe wasting	38.60 ± 5.04	28.73–48.48	
Weight for age			
Normal	57.10 ± 0.76	55.61–58.59	15.10 (0.001)
Moderate underweight	53.455 ± 1.76	50.01–56.91	
Severe underweight	41.20 ± 5.03	31.33–51.06	
Height for age			
Normal	57.17 ± 0.73	55.73–58.60	21.79 (< 0.001)
Moderate stunting	53.19 ± 1.86	49.54–56.83	
Severe stunting	36.67 ± 5.79	25.33–48.01	
CPT			
Yes	57.47 ± 0.68	56.14–58.79	10.6 (0.001)
No	51.06 ± 1.93	47.27–54.85	
ART adherence			
Good	56.97 ± 0.70	55.60–58.35	29.33(< 0.001)
Fair	43.34 ± 2.47	38.49–48.1	
Poor	39.16 ± 4.36	30.61–47.70	
Drug side effect			
Yes	52.26 ± 1.81	48.70–55.81	0.004 (0.95)
No	55.55 ± 0.96	53.67–57.42	
Regimen change			
Yes	45.67 ± 4.36	37.12–54.23	15. 10 (0.000)
No	56.45 ± 0.75	54.99–57.92	

### Predictors of mortality among children on ART

The bivariate Cox proportional hazard regression model indicated that age, WHO clinical stage, hemoglobin level, baseline CD4 count, cotrimoxazole preventive therapy (CPT), nutrition status indicators (weight for height, weight for age, and height for age), ART adherence, and regimen change during follow-up were all associated with survival status (P < 0.05). However, five variables (age, hemoglobin level, baseline CD4 count, and CPT) were associated with HIV mortality for children on ART in multivariable Cox proportional-hazards model. The result of multivariable analysis revealed that children who were less than 1 year were 8.92 times more likely to die as compared to those of older children ≥5 years (AHR; 8.92, 95% CI:2.62–30.36). Children are aged 1–5 years were also 6.42 times more likely to die as compared to children aged ≥5 years (AHR:6.42, 95% CI: 1.40,30.36). Children with a hemoglobin level less than 10 gm/dl were 5.04 times more likely to die as compared to those having hemoglobin level ≥ 10 gm/dl (AHR: 5.04, 95% CI: 1.54–16.55).

Similarly, children having a baseline CD4 count below threshold were 3.55 times more likely to die as compared to those of having a CD4 count above threshold (AHR: 3.55, 95% CI: 1.08–11.67). Children who were not taking cotrimoxazole preventive therapy were at risk of mortality by 2.5 times compared to those who has been taking cotrimoxazole preventive therapy (AHR: 2.5, 95% CI: 1.16–8.20). Furthermore, children who had severe wasting were 5.18 times more likely to die as compared to those who are normal (AHR:5.18, 95% CI: 1.25–21.42) (Table 4).

**Table 4:** Cox regression analysis of predictors of mortality among children on ART at East Gojjam zone public hospitals, Amhara regional state, North West Ethiopia, 2019 (*N* = 251)

Variables	Death *N* (%)	Censored *N* (%)	CHR (95% CI)	AHR (95% CI)	*p*-value
Age					
< 1 year	4 (25)	15 (6.4)	10.57(2.94, 8.02)	**8.92(2.62,30.36)**	**0.000**
1–5 year	6 (37.5)	47 (20.0)	4.54 (1.46, 14.16)	**6.42(1.4, 29.5)**	**0.017**
≥ 5 year	6 (37.5)	173 (73.6)	1	1	
CD4 count					
Below threshold	12 (75)	52 (23.0)	9.27 (2.98, 28.76)	**3.55(1.08, 11.67)**	**0.037**
Above threshold	4 (25)	174 (77.0)	1	1	
Hemoglobin					
< 10 mg/dl	11 68.8)	40(17.2)	9.26(2.98, 28.76)	**5.04(1.54, 16.55)**	**0.008**
≥ 10 mg/dl	5 (31.2)	193 (82.8)	1	1	
Baseline WHO stage					
Stage I &II	6 (37.5)	149 (63.4)	0.36(0.13, − 0.99)	0.45(0.16, 1.28)	0.113
Stage III & IV	10 (62.5)	86 (36.6)	1	1	
Taking CPT					
Yes	5 (31.3)	178 (75.7)	1	1	
No	11 (68.8)	57 (24.3)	4.95 (1.71, 14.30)	**2.5(1.16, 8.20)**	**0.034**
Opportunistic infections					
Yes	10 (62.5)	65 (27.7)	3.78 (1.37, 10.38)	2.18(0.63, 7.57)	0.222
No	6 (37.5)	170 (72.3)	1	1	
Weight for height					
Normal	6 (37.5)	178 (75.7)	1	1	
Moderate wasting	4 (25.0)	52 (22.1)	2.07 (0.58, 7.35)	1.82(0.53, 6.16)	0.336
Sever wasting	6 (37.5)	5 (2.1)	21.39(6.86, 66.72)	**5.18(1.25, 21.42)**	**0.023**
Height for age					
Normal	6 (37.5)	171 (72.8)	1	1	0.996
Moderate stunting	6 (37.5)	56 (23.8)	2.71(0.87, 8.40)	1.01(0.01,241.00)	0.998
Sever stunting	4 (25.0)	8 (3.4)	12.24(3.44, 43.59)	0.70(0.01,1476.91)	0.928
Weight for age					
Normal	6 (37.5)	165 (70.2)	1	1	
Under weight	6 (37.5)	59 (25.1)	2.45 (0.79, 7.59)	2.33(0.54, 6.41)	0.431
Sever under weight	4 (25.0)	11 (4.7)	8.89 (2.49, 31.66)	2.66(0.65, 13.45)	0.311
ART adherence					
Good	8 (50.0)	209 (88.9)	1	1	
Fair	2 (12.5)	17 (7.2)	2.72 (0.58, 12.83)	1.27(0.65, 8.22)	0.847
Poor	6 (37.5)	9 (3.8)	10.89 (3.76, 1.54)	1.49(0.83, 7.54)	0.733
The regimen changed					
No	11 (68.8)	225 (95.7)	6.26 (2.17, 18.07)	5.62(2.34, 82)	0.972
Yes	5 (31.3)	10 (4.3)	1	1	

Values in bold font are used to highlight factors with statistically significant impact on survival

### Test of proportional hazard assumption

Testing the proportional hazard (PH) assumption is vital for interpretation and use of fitted proportional hazard models. Therefore, in this study goodness-of-fit (GOF), particularly the Schoenfeld residuals proportional hazard assumption test for the individual covariates and global tests was used. If P-value < 0.05, then the proportional hazard assumption is rejected. The findings indicated that all variables included in the model satisfied PH assumptions (p-value > 0.05) (Table 5).

**Table 5:** Test of proportional-hazards assumption

Variable	Rho	Chi2	Df	Prob > Chi2
Age	0.03	0.01	1	0.90
Hemoglobin	0.15	0.49	1	0.48
CD4 count	− 0.05	0.07	1	0.79
Weight for height	− 24	0.06	1	0.80
Height for age	0.17	0.06	1	0.81
Weight for age	0.10	0.2	1	0.90
WHO stage	− 0.14	0.37	1	0.54
CPT	0.10	0.17	1	0.68
OI	− 0.22	0.69	1	0.40
ART adherence	0.17	0.61	1	0.44
The regimen changed	− 0.25	1.40	1	0.24
global test		11.96	11	0.37

## Discussion

This study was aimed to assess survival status and predictors of mortality among HIV-positive children on antiretroviral therapy. Socio-demographic, clinical, nutritional and treatment-related determinant of survival were assessed. The study will be an input to policy-makers, program managers, health professionals to estimate survival rate of patients, to decide based on evidence about HIV/AIDS and to support the planning of systems for enhanced HIV/AIDS control and prevention program.

The result of the study showed that at the end of follow-up, 16 children on ART died and 235 children on ART were censored, resulting in a total death prevalence of 6.37% and an incidence rate of 2.56 per 100 child-years-observation. The overall mortality rate observed in this study was consistent with previous studies done in Ethiopia: 2.1% in Wolaita Zone [11], and 2.3% in Zewditu Memorial Hospital [27]. The nearly similar study periods of the two studies mentioned above with our study might be the reason for agreement of results.

However, the overall mortality rate that occurred in this study was lower than other studies conducted in different countries: India (3%) [28], four sub-Saharan Africa countries (5.1%) [29], Malawi (3.4 deaths per 100 patient years (PYs)) [18], South Africa (4.7 deaths per 100 child years) [30], Eastern Ethiopia (3.8 per 1000 child-months) [13], Northwest Ethiopia (4% CYO) [7], and Arba Minich (3.6% CYO) [8]. This discrepancy might be due to difference in the study period as there were changes in treatment modality recently recommended to treat all HIV-positive children regardless of their WHO clinical stage and CD4 count. Another possible reason might be the difference in the stage of HIV/AIDS at ignition of ART. In this study, the majority of the study participants started ART at WHO clinical stage (I and II) but not in others. Children who were initiated ART at early phase had a longer survival time as compared to their counterparts. Furthermore, the differences of the results might be the difference in the duration study. Another possible explanation might be the difference in sample size. There were also studies which reported lower findings; in Nigeria (1%) [31], in Addis Ababa (12.4 deaths per 1000 child-year) [12], and in Mekelle, Ethiopia (1.40 per 1000 child-months) [32].

In this study, age was found to be a significant predictor of mortality among HIV-positive children on ART. The mortality rate was higher among the youngest children less than 5 years than among older children. Children < 1-year-old were 8.92 times more likely to die as compared to children ≥ 5 years old. This finding is consistent with other previous studies conducted in Swaziland [33], India [28], Nigeria [31, 34], Malawi [18], and Ethiopia [9, 10, 27]. Children 1–5 years old were also 6.42 times more likely to die as compared to children ≥ 5 years old. This finding was consistent with another previous study conducted in Ethiopia [9]. This fragility of infants and younger children that end up might have resulted in high mortality rate among children in this age group. Children starting ART before 2 years of age are more likely to have rapid disease progression [31] and this may also be the cause of high mortality in the young children. Further research is needed to identify factors impacting health outcomes among this highly vulnerable group of children. However, this study does not support a study done in Uganda which stated there was no enough evidence to suggest that age had a confounding effect on survival [16].

Children with CD4 count below the threshold level at initiation of ART have higher risk of death (3.55 times) than those children with CD4 count above the threshold. This might be due to the fact that HIV attacks CD4 cells, as a result child with the lower CD4 count could have the chances of acquiring serious diseases which leads to death. This finding is consistent with previous studies conducted in different areas of Ethiopia [6–8, 10, 34]. On the contrary, a study done in Bahir-Dar found that low baseline CD4 cell count was not a predictor of survival time of HIV-infected children [9].

Similarly, in this study the risk of mortality increased among children with low hemoglobin level at baseline. Children with Low hemoglobin level (< 10 gm/dl) were 5.04 times at risk of death as compared with their counterparts. This study was supported by other findings [6–10]. This might be due to the dual effect of anemic condition and some drugs that aggravate anemia which leads to other comorbidity and death.

The findings of present study revealed that malnutrition in the form of severe wasting was a significant predictor of mortality among children on ART. Children who were severely wasted at the time of ART initiation were 5.18 times more likely to die as compared to those who were normal (not wasted) at the time of ART initiation. This finding is consistent with other previous studies [31, 33]. However, baseline nutritional status was not significant predictor of mortality [7].

In this study, cotrimoxazole preventive therapy was found to be an independent predictor of survival that becomes statistically significant in the Cox proportional hazard model. Cotrimoxazole was found to have a protective effect for children on ART (AHR = 2.5,95% CI:1.16 -8.20**).** This finding is consistent with previous findings of different studies [7, 27]. This finding is also supported by WHO recommendation that states all exposed infants and HIV-infected children to start CPT [35].

Unlike most of previous findings [6, 9, 10, 13, 27, 29, 31, 34], it was found that WHO clinical-stages at baseline were not found to be an independent predictors of survival in this study. This discrepancy could be owing to changes in treatment modality recommended to treat all HIV-positive children regardless of their WHO clinical stage and CD4 count. As a result, this cohort reveals a high proportion child were in mild baseline WHO clinical-stages (stages I and II). This study is in line with a previous study done in Ethiopia [7].

## Conclusion and recommendation

This study found that the overall mortality rate was 2.56 per 100 children years observation. The mortality rate was lower when compared with previous studies done in Ethiopia as well as other countries. CD4 count below threshold, children aged < 1 year and 1–5 years, baseline malnutrition in the form of severe wasting (WFH <  − 3z), not taking cotrimoxazole preventive therapy, and baseline Hgb < 10 gm/dl were significant predictors of mortality among HIV-positive children after initiation of ART.

Close monitoring and follow-up should be given for under-five, malnourished and anemic children. Furthermore, the current guideline on the initiation of CPT needs to be revised as children who are not taking CPT are more likely to have lower survival status. It is recommended to conduct prospective cohort studies to better understand the impact of each factor affecting survival status of children on ART.

## Limitations of the study

The findings of this study should be interpreted with consideration of the following conditions: initially the study was a retrospective follow-up study and secondly the number of study subjects was small as compared to the calculated sample size due to the smaller number of children on ART at the two study sites. Moreover, some children who were lost to follow-up were not addressed by tracing mechanism, which may under estimate mortality rate as they might end up in death.

## Data Availability

Please contact the corresponding author for data requests.
